# Genetic Tools in the *Nakaseomyces* clade for Evolutionary Comparisons of Signal Transduction Pathways

**DOI:** 10.1002/yea.70029

**Published:** 2026-06-03

**Authors:** Maria Abraham, Christine L. Iosue, Dennis D. Wykoff

**Affiliations:** ^1^ Department of Biology Villanova University Villanova Pennsylvania USA

**Keywords:** candidiasis, *cis*/*trans* evolution, comparative fungal genomics, promoter evolution, whole genome duplication

## Abstract

The genus *Nakaseomyces* provides four species that are closely related but have different characteristics. For example, *N. glabratus* (formerly known as *Candida glabrata*) is a common human pathogen, whereas *N. bracarensis* and *N. nivariensis* have been isolated in clinical settings but are not common human pathogens. *N. delphensis* was isolated from fruit and there is no evidence it is pathogenic. Given the differences, we developed the clade as a molecular genetic system where we could introduce plasmids and assess transcriptional output from cloned promoters. We engineered a CRISPR/Cas9 plasmid that allows for rapid Gibson cloning of gRNAs, generated auxotrophic strains for amino acids and nucleotides, and introduced plasmids into each species. We used promoter‐YFP plasmids to determine that while there are differences between the species, each species likely has intact thiamine and phosphate (THI and PHO) signal transduction pathways, and that gene expression in *N. glabratus* and *N. bracarensis* is more similar to one another than to the other two species. Finally, we determine that *N. glabratus*, *N. bracarensis*, and *N. nivariensis* persist in a murine macrophage for 24 h, whereas *N. delphensis* does not. This work describes new molecular tools for genetic manipulation in the *Nakaseomyces* clade and allows for evolutionary questions to be explored.

## Summary

- The *Nakaseomyces clade* provides a contrasting set of species to identify how whole genome duplication and gene loss impact niche specialization.
- Using CRISPR/Cas9, four *Nakaseomyces* species are now auxotrophic for various amino acids and uracil, allowing for introduction of *S. cerevisiae* plasmids.
- Three *Nakaseomyces* species persist in mouse macrophages, whereas the one species, *N. delphensis*, that has not been shown to be associated with clinical settings, does not persist.

## 1 Introduction

**Figure 1:**
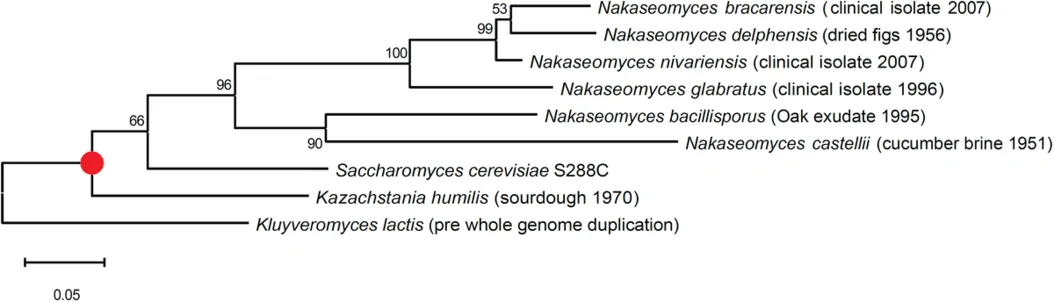
Phylogenetic tree of Ade2 proteins in post‐WGD duplication species along with *K. lactis*. The default parameters for CLUSTALW were used to align those sequences and a maximum likelihood phylogeny was constructed using the default settings in MEGA (v.12) with 1000 bootstrap replicates. The *Nakaseomyces*, *Kazachstania*, and *Saccharomyces* genera all experienced an ancient whole genome duplication, indicated by the red circle, followed by subsequent loss of most of the duplicates.

Pathogenic fungi have become a prevalent cause of infections in hospital settings due to increased use of antibiotics, immunosuppressive treatment, and medical devices. With progress in medicine, fungal pathogens have developed increasing resistance, particularly in immunocompromised individuals, leading to increased incidence of invasive candidiasis (Pappas et al. 2018; Beardsley et al. 2024). Over a million people have systemic fungal infections each year with the majority of these cases leading to death (Denning 2024). *Nakaseomyces glabratus* has risen to be one of the most prevalent causes of candidiasis with an incidence rate almost equal to the well‐known pathogen *Candida albicans* (Askari and Kaur 2024; Beardsley et al. 2024). *N. glabratus* falls within the *Nakaseomyces* “glabrata” clade which includes the nonpathogenic species, *N. delphensis*, and the pathogenic species, *N. nivariensis* and *N. bracarensis*, because of the similarities in their genomes (Figure 1) (Gabaldón et al. 2013; Angoulvant et al. 2015).

This “glabrata” group is intriguing because of the emergence of pathogenicity. Most species in *Saccharomycetales* are not known mammalian pathogens, but three of the four species in the glabrata group are mammalian pathogens (Angoulvant et al. 2015; Askari and Kaur 2024). Additionally, the likely ancestor was not pathogenic, and the three pathogenic species have different gene expansions (*EPA* and *YPS* gene families) related to pathogenicity (Gabaldón et al. 2013; Marcet‐Houben et al. 2024), suggesting that pathogenicity may have arisen multiple times.

The other intriguing thing about this group is that it is an example of how species resolve a whole genome duplication event in different ecological niches. Along with *Saccharomyces cerevisiae*, the *Nakaseomyces* clade is part of a post‐whole genome duplication group which likely has a lineage from a tetraploid ancestor that underwent whole genome duplication (WGD) due to hybridization between species (Wolfe 2015). The *Saccharomyces, Nakaseomyces*, and *Kazachstania* genera all shared a common ancestor that experienced a whole genome duplication event, whereas *Kluyveromyces* and other genera did not experience the WGD (Wolfe and Shields 1997; Wolfe et al. 2015; Devillers et al. 2022; Opulente et al. 2024).

Certain defining characteristics of the *Nakaseomyces* clade have been determined through previous research (Gabaldón et al. 2013; Legrand 2016; Gabaldón and Fairhead 2018). The species are (1) haploid, allowing for simple genotype to phenotype observations, (2) grow in standard *S. cerevisiae* medium, (3) are Crabtree‐positive (generate alcohol in the presence of oxygen), and (4) some have been genetically manipulated (Zhou Li et al. 2021; Métivier et al. 2024). Having a genetically manipulatable clade of species is reminiscent of studies with *Drosophila*, where the different behaviors and characteristics of that genera can be exploited to understand more complex evolutionary biology (O'Grady and DeSalle 2018). We undertook this work to generate a genetically tractable clade to ask evolutionary questions such as: are newly evolved promoters functional in species that have never seen these promoters? Our previous work with the phosphate and thiamine starvation inducible phosphatases *NgPMU2* and *NgPMU3* suggests that the common ancestor of these species did not have the appropriate *trans* factors to regulate newly evolved phosphate and thiamine regulated promoters (Kerwin and Wykoff 2012; Nahas et al. 2018).

Here, building on previous work from other laboratories where they transformed these species and generated auxotrophic strains, we have developed additional new molecular tools based on a modified CRISPR/Cas9 plasmid and used it in the *Nakaseomyces* clade (Enkler et al. 2016; Zhou Li et al. 2021; Métivier et al. 2024). In an effort to expand on these molecular genetic tools, we generated auxotrophic strains for adenine, histidine, uracil, and leucine, sequenced the genomic locus for a subset of the auxotrophic strains, confirmed the predicted growth characteristics of individual species, introduced plasmids with a variety of selectable markers, and expressed various promoters driving YFP expression. Finally, we confirmed that *N. delphensis*, which has not been isolated as a known pathogen, is less able to persist in mouse macrophages. We believe that this work helps to develop the *Nakaseomyces* clade as a genetically manipulatable system allowing for comparative studies between *S. cerevisiae* and the four *Nakaseomyces* species.

## 2 Materials and Methods

### 2.1 Yeast Strains and Growth Medium

Yeast strains used in this study are listed in Supporting Table 1. Strains were grown in yeast extract, peptone, dextrose (YEPD) medium or synthetic dextrose (SD) medium supplemented with complete supplement mixture (CSM) with or without histidine, uracil, leucine, or adenine, depending on the experiment. All SD and CSM combinations were purchased from Sunrise Science, Tennessee.

### 2.2 Growth Conditions

**Figure 2:**
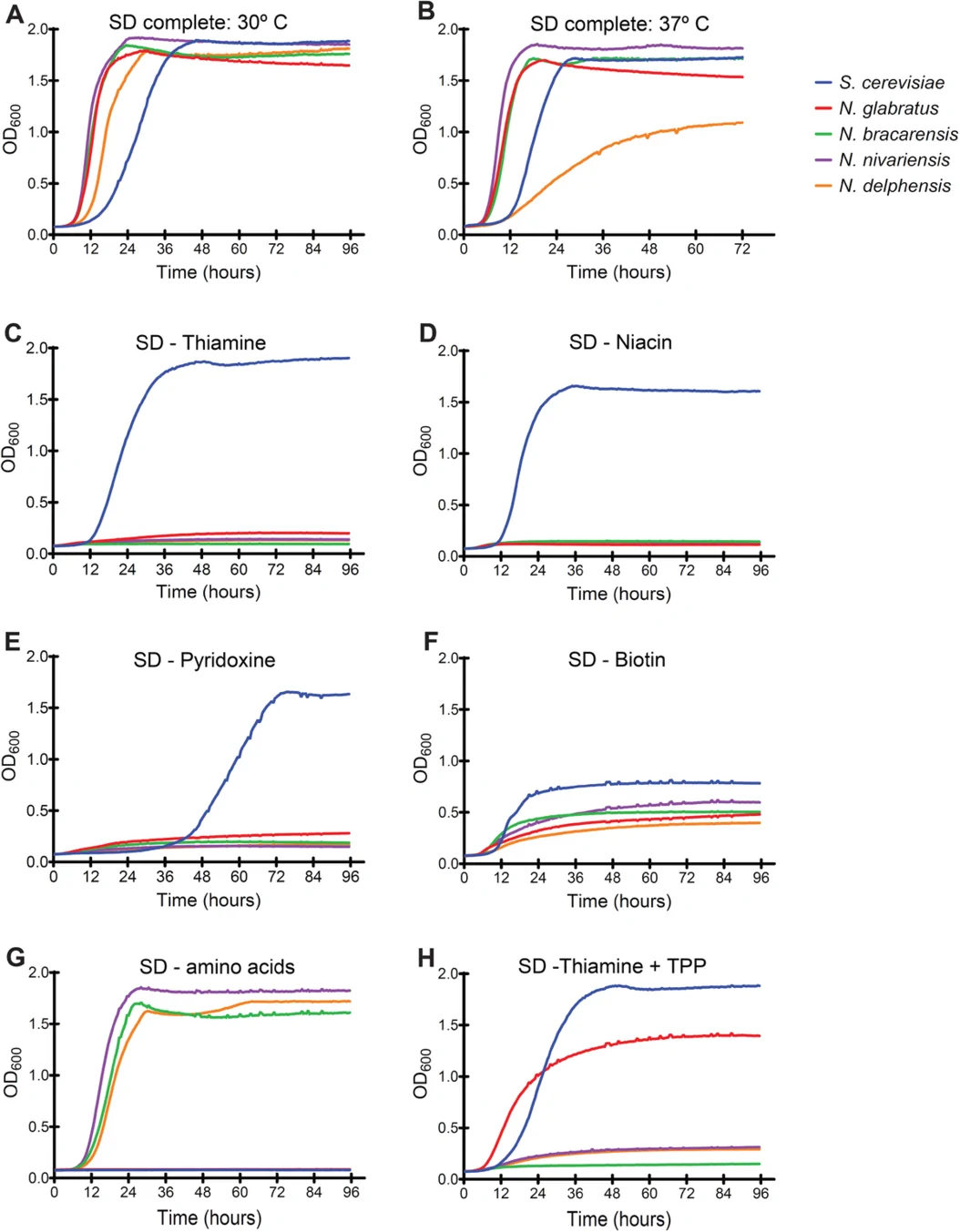
Growth of *S. cerevisiae* and the *Nakaseomyces* species in various conditions. Strains were grown in synthetic dextrose (SD) medium lacking B vitamins or amino acids at 30°C (A, C–H) or 37°C (B) and optical density (OD_600_) was measured every 0.5 h for 72–96 h with a CLARIOstar plate reader. Lines represent the mean of three replicates for each species.

To assess growth in different conditions, yeast strains were first grown overnight at 30°C in SD complete medium, then cells were pelleted and washed three times with sterile water. Cells were inoculated into SD complete or SD lacking different B vitamins, specifically thiamine, niacin, pyridoxine, and biotin, or lacking amino acids. Strains were grown in a 96 well plate at a starting optical density (OD_600_) of 0.001. Optical density was measured every 0.5 h for 72–96 h with a CLARIOstar plate reader (BMG Labtech) and plotted as a line graph, with each line representing the mean of three replicates. Strains were always grown at 30°C, except in Figure 2B, where growth was measured at 37°C. To assess growth of the wild‐type and auxotrophic strains, yeast strains were first grown on solid YEPD medium, then replica‐plated to solid YEPD with nourseothricin (100 µg/mL), SD‐uracil, SD‐histidine, and SD‐adenine.

### 2.3 Cloning gRNA Sequences Into CRISPR/Cas9 Plasmid

To utilize CRISPR/Cas9 in the *Nakaseomyces* species, we modified pV1435 from the Gerald Fink laboratory (Addgene plasmid #111439) (Vyas et al. 2018) to have a *Pac*I site upstream of the gRNA sequence using PCR (primer sequences are listed in Supporting Table 2) and Gibson assembly (New England Biolabs). The presence of the *Pac*I site was confirmed through PCR, sequencing of the locus, and digestion with *Pac*I restriction enzyme. Using a lithium acetate transformation protocol, the modified CRISPR/Cas9 plasmid was transformed into *N. glabratus* to confirm that the gRNA targeting *NgADE2* was still functional.

To integrate a new gRNA sequence into this plasmid, primers containing the new gRNA were annealed and repaired into the *Pac*I digested plasmid using Gibson assembly. PCR was used to confirm the addition of the new gRNA sequence as well as the loss of the original gRNA targeting *NgADE2*. We identified between 1 and 10 auxotrophic strains for the different species and have included the success rate (ranging from 0% to 100% success) of individual gRNAs in generating auxotrophic strains in the Supporting Table 3.

### 2.4 Using CRISPR/Cas9 to Generate Auxotrophic Yeast Strains

To make auxotrophic strains of the *Nakaseomyces* species, gRNA sequences targeting *LEU2* or *ADE2* in *N. glabratus* and *HIS3* in *N. bracarensis*, *N. nivariensis*, and *N. delphensis* were integrated into the CRISPR/Cas9 plasmid we engineered. The plasmid was transformed into the *Nakaseomyces* species using a standard lithium acetate transformation protocol and transformants were selected on YEPD with nourseothricin (100 µg/ml). We confirmed a lack of growth on solid SD medium lacking leucine, adenine, or histidine. PCR and sequencing of the targeted locus demonstrated frameshift mutations in the genes.

### 2.5 Plasmids

*S. cerevisiae* ARS/CEN plasmids were used to introduce genes or promoter‐gene fusions into alternate yeast species. To assess growth after addition of a gene from another species, the ORF of the gene of interest, as well as a highly expressed promoter, was amplified by PCR and repaired into a digested *HIS3* plasmid (pRS313) using homologous recombination in yeast (Corrigan 2013). The presence of the promoter and gene was confirmed by PCR, sequencing, and function in the native species. These plasmids were then transformed into the other *Nakaseomyces* species using a standard lithium acetate transformation protocol and selection on SD‐histidine.

To measure expression of *S. cerevisiae* and *Nakaseomyces* promoters, we constructed plasmids where 1 kbp promoter (where ‐1 is the nucleotide upstream from the start codon) of the gene of interest was transcriptionally fused to YFP, allowing expression to be measured via fluorescence. The promoter sequence was amplified by PCR and repaired into a digested pRS313 plasmid containing YFP as previously described. The presence of the promoter was confirmed by PCR, sequencing, and function in the native species. These promoter‐YFP plasmids were then transformed into the other *Nakaseomyces* species and selected for on SD‐histidine plates.

### 2.6 Flow Cytometry

To assay the expression of promoters, yeast strains containing promoter‐YFP plasmids were grown overnight at 30°C in SD‐histidine medium. Cells were pelleted, washed three times with sterile water, and inoculated into SD‐histidine medium for *ADH1* induction. For *THI4* and *NgPMU3* induction, strains were grown in SD‐histidine medium without thiamine or with thiamine supplemented (3 mg/L; high thiamine). For *NgPMU2* induction, strains were grown in SD‐histidine medium without phosphate or with phosphate supplemented (10 mM KH_2_PO_4_; high phosphate). All strains were grown overnight (~18 h) at 30°C in triplicate. A flow cytometer with a 533/30 filter set (Accuri C6 Plus, BD Biosciences) was used to measure the fluorescence of 10,000 cells for each sample. Mean fluorescence (in arbitrary units) was reported.

### 2.7 Macrophage Persistence Assay

A previously characterized persistence assay in mouse macrophages (Pezak et al. 2024) was used to determine the ability of *Nakaseomyces* species to survive in macrophages as a proxy for pathogenicity. J774.A1 mouse macrophage cells were cultured in DMEM+Glutamax (Gibco), supplemented with 10% FBS, sodium bicarbonate, and 1x antibiotic‐antimycotic (Gibco catalog #15240062, Thermo Fisher), at 37°C in a 5% CO_2_ containing incubator. Macrophages were grown to ~90% confluency, cells were harvested and 0.5 mL of this cell suspension was seeded into a 24 well plate and grown for 24 h at 37°C in 5% CO_2_. For incubation with yeast cells, media was removed and the macrophage cells were washed once before adding 0.5 mL warmed DMEM+Glutamax but **without** antibiotic‐antimycotic. Yeast cell numbers were quantified by flow cytometry prior to incubation and 100,000 yeast cells were added to each well and gently mixed by pipetting. The 24 well plate was centrifuged for 1 min at 1000 rpm. After 1 h at 37°C in 5% CO_2_, media was removed and replaced with DMEM+Glutamax, with antibiotic‐antimycotic for 1 h. Wells corresponding to 2 h were washed with DMEM+Glutamax, with 10% FBS and sodium bicarbonate but **without** antibiotic‐antimycotic and lysed with 0.1% Triton X‐100 by scraping with a pipette tip. The lysed sample was plated on YEPD petri plates, grown 1–2 days at 30°C, and yeast colonies were counted. DMEM+Glutamax, with 10% FBS and sodium bicarbonate but **without** antibiotic‐antimycotic was added to wells corresponding to 24 h after infection and the plate was incubated at 37°C in 5% CO_2_ for an additional 22 h. Wells were washed and lysed as described and plated on YEPD petri plates and yeast colonies were counted. We confirmed that a 1‐h treatment in DMEM+Glutamax, with 10% FBS and sodium bicarbonate **with** antibiotic‐antimycotic, had no effect on viability. Data is presented as a bar for both the initial 2 h and final 24 h timepoint, with each bar representing the mean and standard deviation of three replicates.

## 3 Results

### 3.1 Characterization of Growth Characteristics of N. glabratus, N. bracarensis, N delphensis, and N. nivariensis

Using genomic BLAST algorithms, we confirmed the results of others that *N. glabratus, N. bracarensis, N delphensis*, and *N. nivariensis* do not contain genes required for the synthesis of the B vitamins B1 (thiamine), B3 (niacin), B6 (pyridoxine), and B7 (biotin) (Domergue et al. 2005; Gabaldón et al. 2013). While lacking critical biosynthetic genes, we have found that analogs can substitute for missing genes – *CgPMU2* can functionally replace the *ScPHO5* gene (Orkwis et al. 2010), raising the possibility of cryptic genes substituting for known genes. Thus, we wished to verify that the species are unable to grow in the absence of these vitamins. Figure 2 presents data that confirms the genomic predictions. First, we demonstrated that all species grow in synthetic medium with 12 amino acids, adenine, and uracil supplied at both 30°C as well as at 37°C, the temperature which mammalian pathogens need to grow (Figure 2A,B). Whereas *S. cerevisiae* is able to grow in the absence of thiamine, niacin, and pyridoxine (Figure 2C–E), none of the *Nakaseomyces* species can grow. Because so little biotin is required for growth, we see evidence of more growth in all species non‐specifically (Perli et al. 2020). Our laboratory strain of *S. cerevisiae* is a biotin auxotroph (Hall and Dietrich 2007), and all species appear to grow the same, but stop growing after biotin is exhausted (Figure 2F). Our laboratory strains of *N. glabratus* and *S. cerevisiae* are auxotrophic for the synthesis of amino acids or uracil (*Nghis3ura3* and *Schis3ura3leu2trp1*), and thus they serve as controls to demonstrate that wild‐type *Nakaseomyces* is able to grow in the absence of 12 amino acids, adenine, and uracil that is supplied in CSM supplement (Figure 2G).

We consistently observe a delay in growth in pyridoxine starvation in *S. cerevisiae*, and this is explained by a delay in the induction of *ScSNO/SNZ* genes (Paxhia and Downs 2018). We also included growth characteristics for strains when TPP (thiamine pyrophosphate) is supplied as the sole thiamine source (Figure 2H). These data demonstrate that among the four *Nakaseomyces* species, only *N. glabratus* has an external phosphatase able to scavenge thiamine. We have previously determined this is a consequence of a tandem duplication present only in *N. glabratus* of the *PMU1‐3* locus (Iosue et al. 2016; Nahas et al. 2018; Iosue et al. 2020).

### 3.2 Generation of Auxotrophs in Nakaseomyces Species

Using a lithium acetate transformation with all of the species (Gietz and Schiestl 2007; Zhou Li et al. 2021) we observed efficient transformation of > 100 cfu with an ARS/CEN plasmid containing a nourseothricin resistance cassette and nourseothricin (100 ug/mL) (Sikorski and Hieter 1989). We confirmed that 2 µ based plasmids were not maintained in any of the *Nakaseomyces* species (only unstable microcolonies form), but *S. cerevisiae* ARS/CEN plasmids were maintained with selection (Zhou et al. 1994). No nourseothricin resistant colonies formed in the absence of transforming DNA for all species except *N. bracarensis*, which does form a few colonies. We also confirmed previous work that *N. delphensis* requires a lower heat shock treatment, which increases transformation efficiency (Zhou Li et al. 2021). To begin the process of generating auxotrophic strains, we generated stable *ura3* mutants by selection on 5‐flouroorotic acid containing plates.

**Figure 3:**
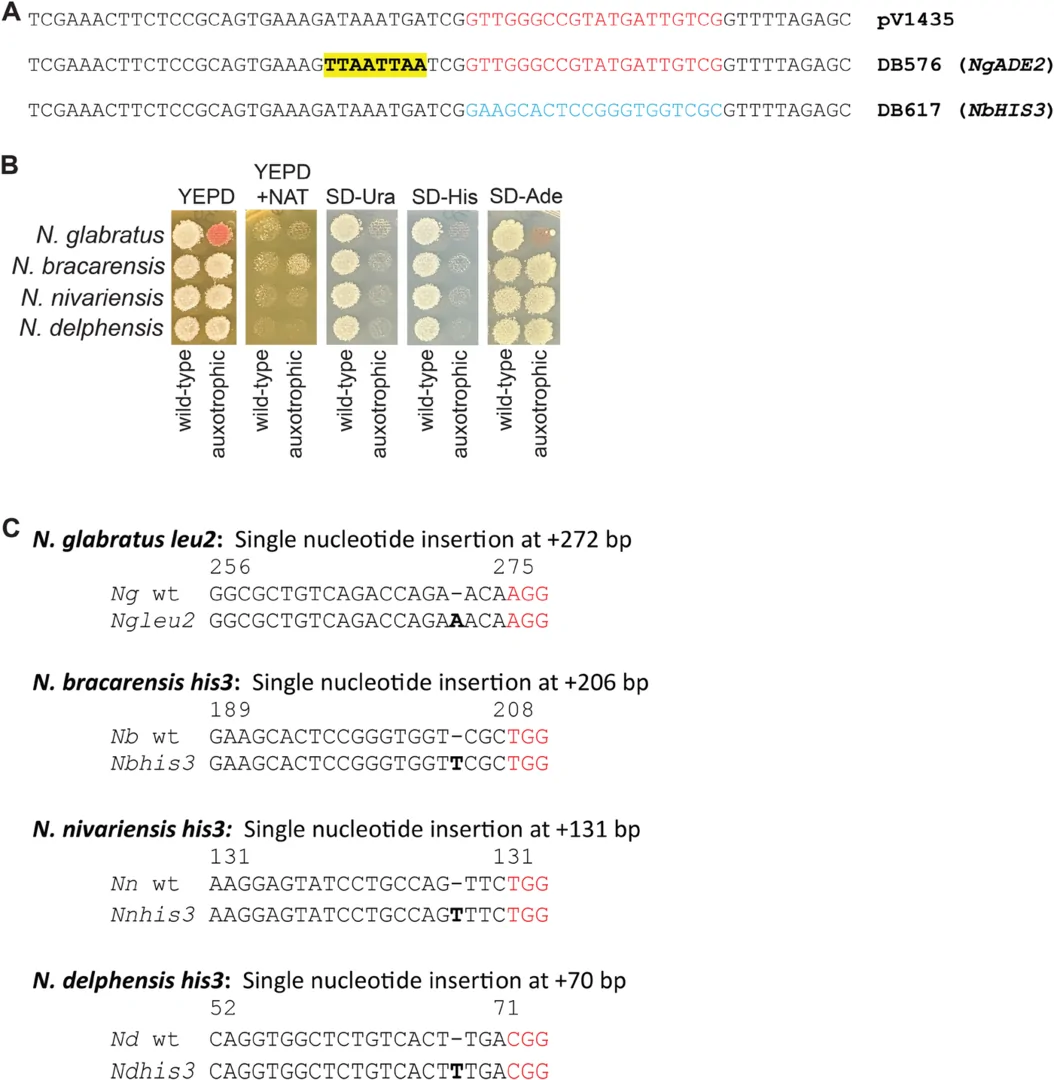
Generation of auxotrophs in *Nakaseomyces* species using CRISPR/Cas9. (A) A modified form of the pV1435 plasmid from the Fink laboratory (Vyas et al. 2018) was used to engineer a CRISPR/Cas9 plasmid with a *Pac*I site (highlighted) just upstream of the *NgADE2* gRNA (red text), which allowed for integration of different gRNA sequences (blue text) into the plasmid. (B) This CRISPR/Cas9 plasmid was used to generate *his3ura3* auxotrophs in the *Nakaseomyces* species, which are sensitive to nourseothricin (100 ug/ml). With *N. glabratus*, we generated *ade2his3ura3* and *leu2his3ura3* triple mutants (*leu2his3ura3* not shown). (C) Frameshift mutations in the *leu2* mutants of *N. glabratus* and *his3* mutants of the *Nakaseomyces* species were confirmed by PCR and sequencing of the loci. Only single nucleotide insertions were identified, and the numbers indicate the location from the ATG start site. Red text denotes the NGG sequence. Nucleotide in bold indicates the single nucleotide insertion.

Next, we engineered a new CRISPR/Cas9 plasmid that allowed for rapid cloning of new gRNAs and selection in the *Nakaseomyces* species (Figure 3A). The plasmid is a modified form of pV1435 from the Fink laboratory (Vyas et al. 2018), with 3 nucleotides changed to engineer a *Pac*I site (highlighted in Figure 3A) just upstream of the gRNA, allowing for primer design and Gibson cloning of the gRNA into the plasmid (Gibson et al. 2010). The added benefit of this modified plasmid is that it contains a gRNA targeting the *NgADE2* gene (red text in Figure 3A) which allows for a parallel red/white screen of transformation efficiency in *N. glabratus*, as has been previously demonstrated (Enkler et al. 2016; Métivier et al. 2024). After cloning sequence optimized gRNAs, using CHOP/CHOP to identify targets (Labun et al. 2019), transformation is straight forward with a selection for either nourseothricin resistance or uracil prototrophy.

We found with all *Nakaseomyces* species that the selection for uracil prototrophs was leaky, meaning that the selection generally only works with low cell density plating. When more than 10^6^ cells were plated, transformants did not form clear colonies, requiring multiple replica plating steps to dilute non transformed cells. We assume that this may be a consequence of dead cells providing a source of uracil so that cells can grow slowly even when they are not uracil prototrophs. Because of this, we used the resistance to nourseothricin to select for the presence of the CRISPR/Cas9 plasmid, except with *N. bracarensis*, where we observed some nourseothricin resistant colonies even when DNA was not included in the transformation. In all cases, we chose two gRNAs near the 5' end of the ORF for each gene and at least one of the two gRNAs (see Supporting Table 2 for sequences) was successful in producing auxotrophic strains (Figure 3B). We generated auxotrophic strains and double auxotrophic strains. With *N. glabratus*, we also generated *ade2his3ura3* and *leu2his3ura3* triple mutants.

Using this CRISPR/Cas9 tool, we generated *leu2* mutants of *N. glabratus* and *his3* mutants of the *Nakaseomyces* species and confirmed frameshift mutations in each species by PCR and sequencing of the loci. We identified only single‐nucleotide indels and plated > 10^7^ cells on medium lacking the appropriate amino acid to identify mutants that reverted rarely that is zero or 1‐2 colonies per plate. The sequences of CRISPR/Cas9 generated auxotrophic mutants are presented in Figure 3C.

### 3.3 ARS/CEN Plasmids Designed for S. cerevisiae Are Effective for Transformation Into Nakaseomyces

Using *S. cerevisiae* derived ARS/CEN containing plasmids and lithium acetate transformation allows for 20‐500 colonies per transformation when we select for uracil, histidine, or leucine prototrophy, using the *URA3*, *HIS3*, or *LEU2* genes in the pRS300 series of plasmids (Sikorski and Hieter 1989). However, the transformation efficiency per µg of DNA is between 5 and 10‐fold lower in *N. bracarensis*, *N. delphensis*, and *N. nivariensis* relative to *N. glabratus* or *S. cerevisiae*.

**Figure 4:**
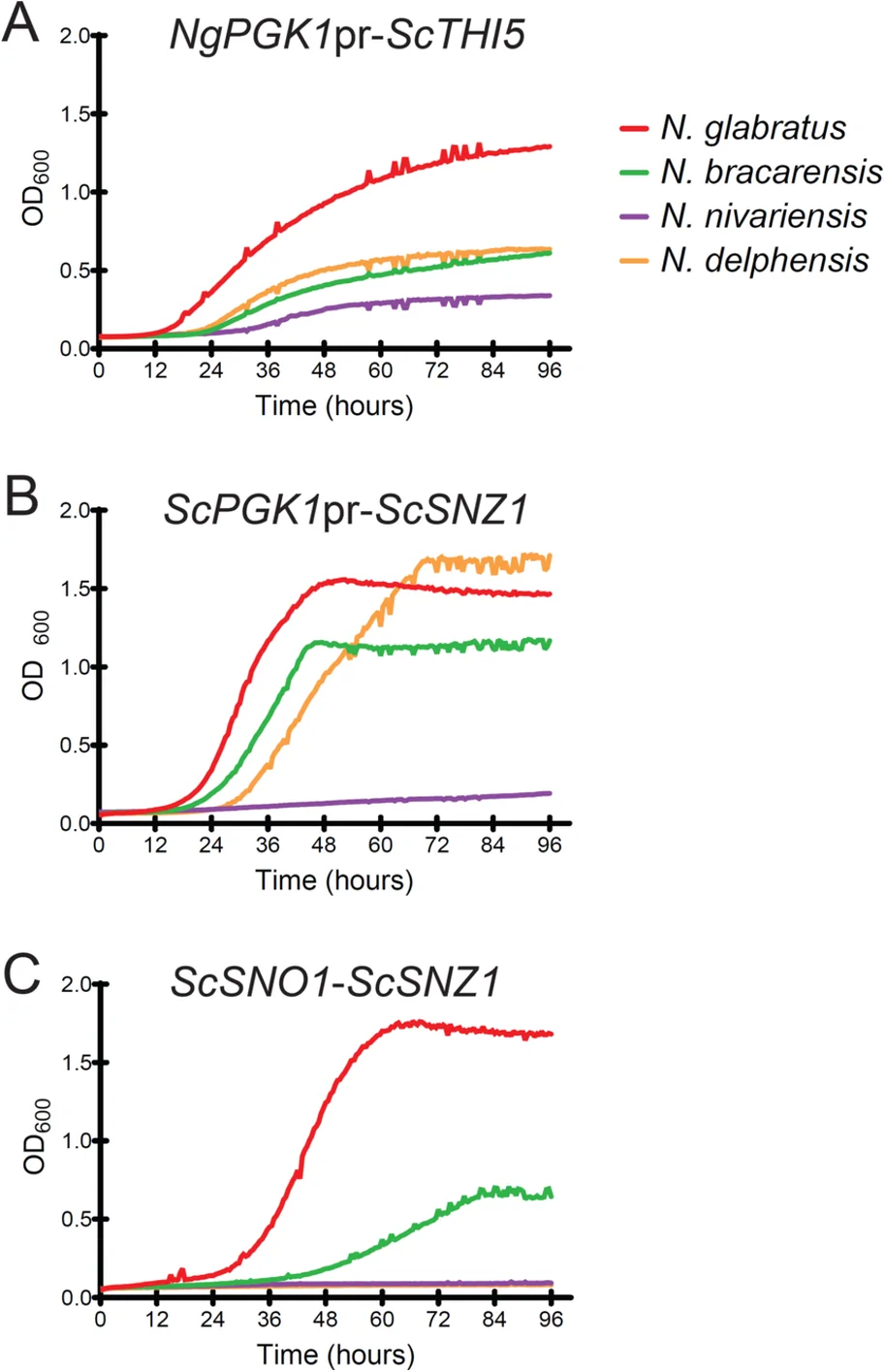
Plasmids containing *S. cerevisiae* genes can be transformed and expressed in the *Nakaseomyces* species. (A) *ScTHI5*, (B) *ScSNZ1*, and (C) *ScSNO1/ScSNZ1* were cloned into plasmids under the control of different promoters, transformed into the *Nakaseomyces* species, and grown in the absence of thiamine (A) or pyridoxine (B, C). Optical density (OD_600_) was measured every 0.5 h for 96 h with a CLARIOstar plate reader. Lines represent the mean of three replicates for each species.

To determine if heterologous genes can be expressed from these plasmids, we introduced plasmids containing *ScTHI5* or *ScSNZ1/SNO1* and assessed their ability to confer growth in the absence of thiamine or pyridoxine in some *Nakaseomyces* species (Figure 4). We had previously determined that overexpression of *ScTHI5* with the *NgPGK1* promoter confers thiamine prototrophy to *N. glabratus*, and hypothesized that expression of *ScSNZ1/SNO1* would confer pyridoxine prototrophy (Iosue et al. 2016; Paxhia and Downs 2018). Overexpression of *ScTHI5* confers the ability to grow in the absence of thiamine in all of the species at varying rates (Figure 4A), and overexpression of *ScSNZ1* confers pyridoxine prototrophy, with *N. nivariensis* growing very slowly (Figure 4B). We also introduced the *ScSNO1‐SNZ1* gene cluster under the control of the native *S. cerevisiae* promoters and assessed growth in the absence of pyridoxine (Figure 4C). These data indicate that we can express gene products from *S. cerevisiae* and confer prototrophy to varying degrees, depending on promoter strength.

Whereas plasmids appear stable enough in *N. glabratus*, *N. bracarensis*, and *N. delphensis* to confer prototrophy, *N. nivariensis* grows very slowly in medium lacking pyridoxine even when we express *ScSNZ1* under the control of a highly expressed promoter. This could be a consequence of poor plasmid maintenance in this species, or poor expression from the promoter, as even in the absence of thiamine *N. nivariensis* grows poorly with *ScTHI5* under the control of the *NgPGK1* promoter.

### 3.4 Expression of Promoter‐YFP Plasmids in Nakaseomyces Indicates Transcription is Optimized for Species

**Figure 5:**
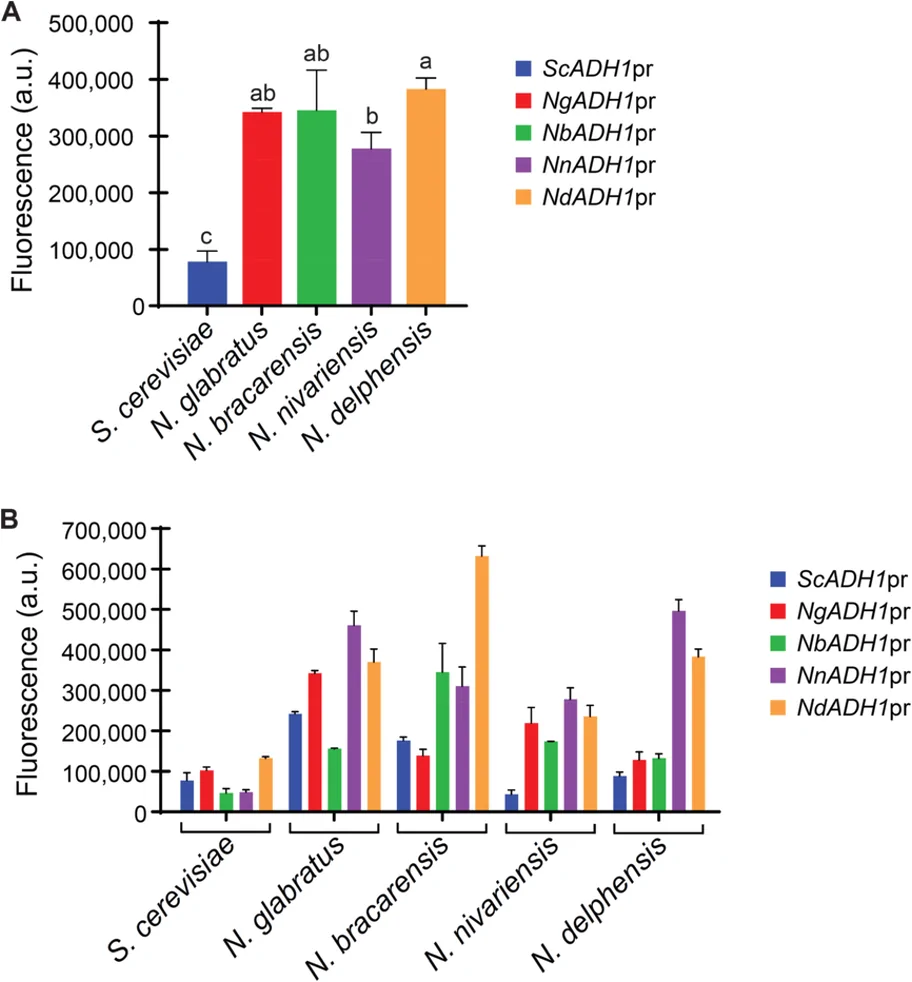
Expression of the *ADH1* promoter in *Nakaseomyces* species. 1 kbp *ADH1* promoter from each *Nakaseomyces* species was transcriptionally fused to YFP in a plasmid. Each species' *ADH1* promoter was transformed into the matching species, i.e., *NgADH1*pr‐YFP into *N. glabratus* (A) and transformed into the other *Nakaseomyces* species (B). Strains were grown in SD medium without histidine and mean fluorescence was measured using a flow cytometer. Bars represent the mean and standard deviation of three replicates. A one‐way ANOVA with a post hoc Tukey's multiple comparisons test was performed and is reported as a compact letter display in (A). Samples that share the same letter are not statistically different from each other.

To dissect the contribution of plasmid stability and optimized promoter expression, we generated plasmids that contained 1 kbp promoters in front of the gene encoding yellow fluorescent protein (YFP), which allowed us to use flow cytometry to quantify expression of promoters. First, we cloned the *ADH1* promoter from each species into a *HIS3* plasmid containing a transcriptional fusion of YFP and selected for plasmid maintenance by growing the cells in the absence of histidine. The *ADH1* promoter from each species expressed approximately at the same level when in its matching species (Figure 5A, Figure S1), suggesting plasmids are likely equally stable in each species and promoters are capable of high‐level expression. We have included *ScADH1* promoted YFP as well and observed lower expression in *S. cerevisiae*. This lower expression is likely a consequence of plasmid abundance. The plasmids we use are optimized for segregation in *S. cerevisiae*, and the only species that exhibits bimodal YFP expression is *S. cerevisiae* (Figure S1). Thus, it seems likely that the defect we observed in *N. nivariensis* growth with *ScTHI5* and *ScSNZ1* is a consequence of low expression from the *NgPGK1* or *ScPGK1* promoters expressing these ORFs.

To further investigate expression of alternate promoters in these species, we transformed plasmids containing a different species' *ADH1* promoted YFP into the *Nakaseomyces* species and measured expression in SD medium lacking histidine (Figure 5B). The results indicate that while all promoters appear to function, there is variability of expression between species, and few *Nakaseomyces* promoters express as highly in *S. cerevisiae*. However, this is likely due to *S. cerevisiae* plasmid maintenance, where functional autonomously replicating sequences and centromeres impose tight copy number control, typically resulting in only 1–2 copies per cell (Sikorski and Hieter 1989). Our purpose in generating these data are to determine if promoters are functional. We are hesitant to make conclusions on actual expression levels, as these interpretations are influenced by each individual species' *cis*‐*trans* interactions at the promoter.

**Figure 6:**
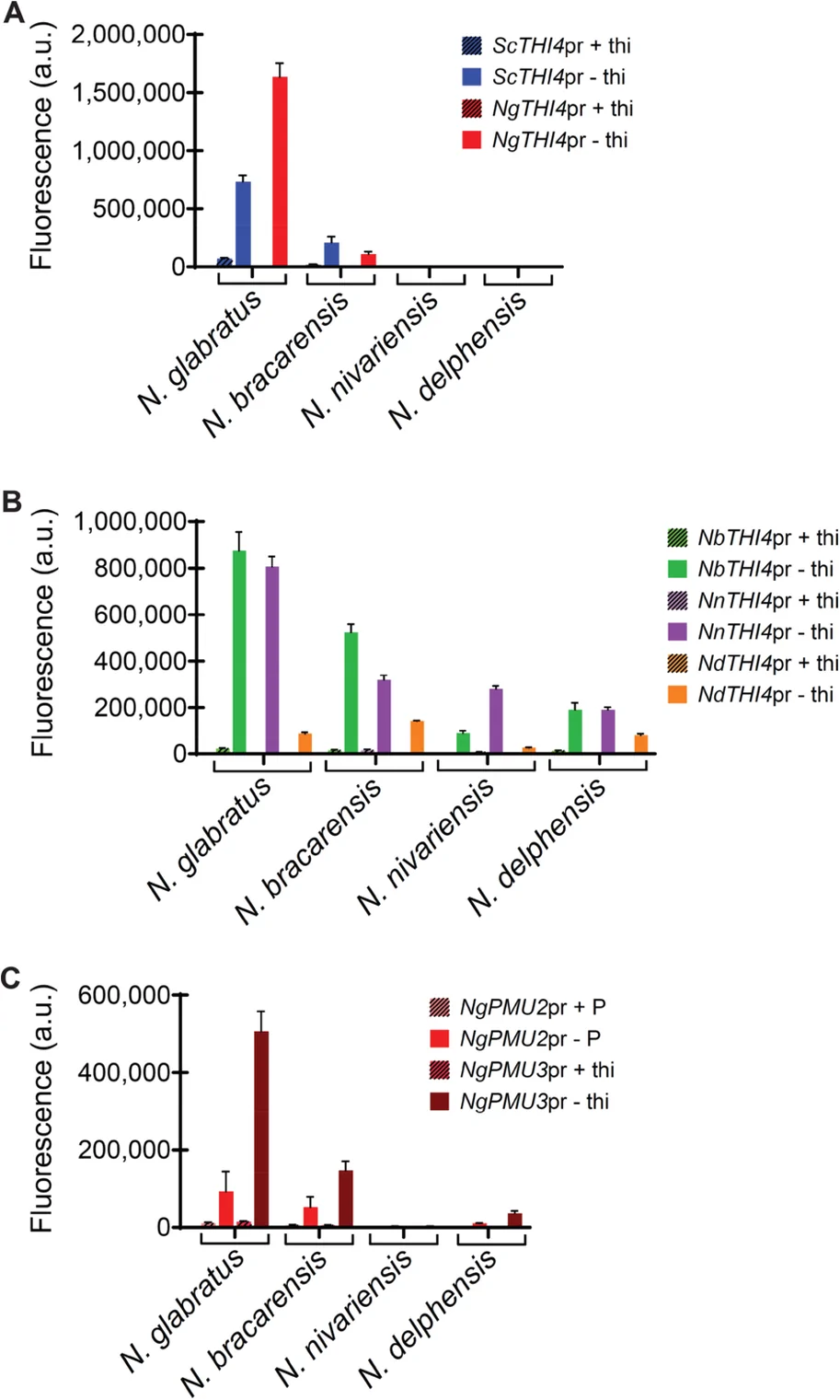
Expression of thiamine and phosphate regulated promoters in *Nakaseomyces* species. *THI4* promoter from *S. cerevisiae* and *N. glabratus* (A) and *N. bracarensis*, *N. nivariensis*, and *N. delphensis* (B) was fused to YFP in a plasmid and transformed into the *Nakaseomyces* species. Strains were grown in SD medium without histidine in high thiamine and thiamine starvation conditions. (C) *PMU2* and *PMU3* promoters from *N. glabratus* were fused to YFP in a plasmid and transformed into the *Nakaseomyces* species. Expression of *NgPMU2* promoter was measured in SD medium without histidine in high phosphate and phosphate starvation conditions. Expression of *NgPMU3* promoter was measured in SD medium without histidine in high thiamine and thiamine starvation conditions. Mean fluorescence was measured using a flow cytometer. Bars represent the mean and standard deviation of three replicates.

*Nakaseomyces* species have partial thiamine biosynthetic pathways and only *N. glabratus* has a new phosphate regulated promoter (*NgPMU2*). This raised the question of whether we could dissect promoter regulation by examining expression in a range of species. To determine expression levels of thiamine‐regulated promoters, we constructed *THI4* promoted‐YFP plasmids and introduced them into the matching species and other species to look for trends (Figure 6). *ScTHI4* and *NgTHI4* promoters express at a high level in *N. glabratus* and to a lesser extent in *N. bracarensis* (Figure 6A). *THI4* is tightly regulated by external thiamine concentrations, suggesting that there is a thiamine signal transduction (THI) pathway that senses thiamine starvation and induces a transcriptional response, even though these species are unable to synthesize thiamine. We observed very low expression of the *ScTHI4* and *NgTHI4* promoters in *N. nivariensis* and *N. delphensis* (the mean fluorescence values for these two species ranges from ~100 to 3000 a.u.), which is consistent with those two species being more closely related to one another (Gabaldón et al. 2013; Opulente et al. 2024). The results with *N. nivariensis* and *N. delphensis* indicate either there is no THI pathway in these two species, or *ScTHI4* and *NgTHI4* specifically do not function in the THI pathway.

To confirm whether there was a functioning THI pathway in *N. nivariensis* and *N. delphensis*, we examined expression of the *THI4* promoter from these species (Figure 6B). *NnTHI4* and *NdTHI4* promoters are upregulated in response to thiamine starvation in the native species, as well as in *N. glabratus* and *N. bracarensis*. *NbTHI4* showed a high level of expression in *N. bracarensis* as well as in *N. glabratus*, while exhibiting a lower level of expression in *N. nivariensis* and *N. delphensis*.

Examining the trends in promoter expression in the four *Nakaseomyces* species allows us to conclude that there are functional THI pathways in all of the species, promoters are optimized for each species, and that for some reason *N. glabratus* expresses promoters better than the other *Nakaseomyces* species, as measured by seeing more fluorescence for a a promtoer YFP construct in *N. glabratus* relative to other species. One caveat is that we are measuring YFP fluorescence, and so there could be post‐transcriptional processes that lead to an apparent elevated fluorescence.

In the *Nakaseomyces* clade, only *N. glabratus* has an external phosphate‐regulated acid phosphatase, encoded by *NgPMU2* (Orkwis et al. 2010), and an external thiamine‐regulated thiamine pyrophosphatase, encoded by *NgPMU3* (Nahas et al. 2018; Iosue et al. 2020). However, all of the species appear to contain *PHO4*, the gene encoding the phosphate starvation regulated transcription factor (Kerwin and Wykoff 2009; Kerwin and Wykoff 2012; He et al. 2017), and *PDC2*, the gene encoding the thiamine starvation regulated transcription factor (Iosue et al. 2023; Dottor et al. 2024). We asked the question of whether a new promoter, *NgPMU2* or *NgPMU3*, would still be regulated by the PHO or THI pathway in the other *Nakaseomyces* species, given that the organisms had not evolved with that promoter in its genome.

We transformed the *NgPMU2* or *NgPMU3* promoter‐YFP plasmids into the four *Nakaseomyces* species and queried whether the promoters were regulated by phosphate or thiamine and to what extent the promoters were upregulated during starvation (Figure 6C). We observe that both promoters in *N. bracarensis* behave similar to *N. glabratus*, suggesting that *N. bracarensis* still has the appropriate *trans* regulation to recognize the promoters. Conversely, *N. nivariensis* is unable to upregulate the promoters, whereas *N. delphensis* is able to regulate the promoters, but only to a small extent. These data suggest that more evolutionary distance between species eliminates the ability to recognize the *cis* element appropriately, and is consistent with the new promoters also not being recognized by *S. cerevisiae* when they are only 1 kbp in length (Kerwin and Wykoff 2012; Iosue et al. 2023).

### 3.5 *N. glabratus*, *N. bracarensis*, and *N. Nivariensis* Are Able to Persist in a Macrophage Survival Model, but *N. delphensis* Is Not

**Figure 7:**
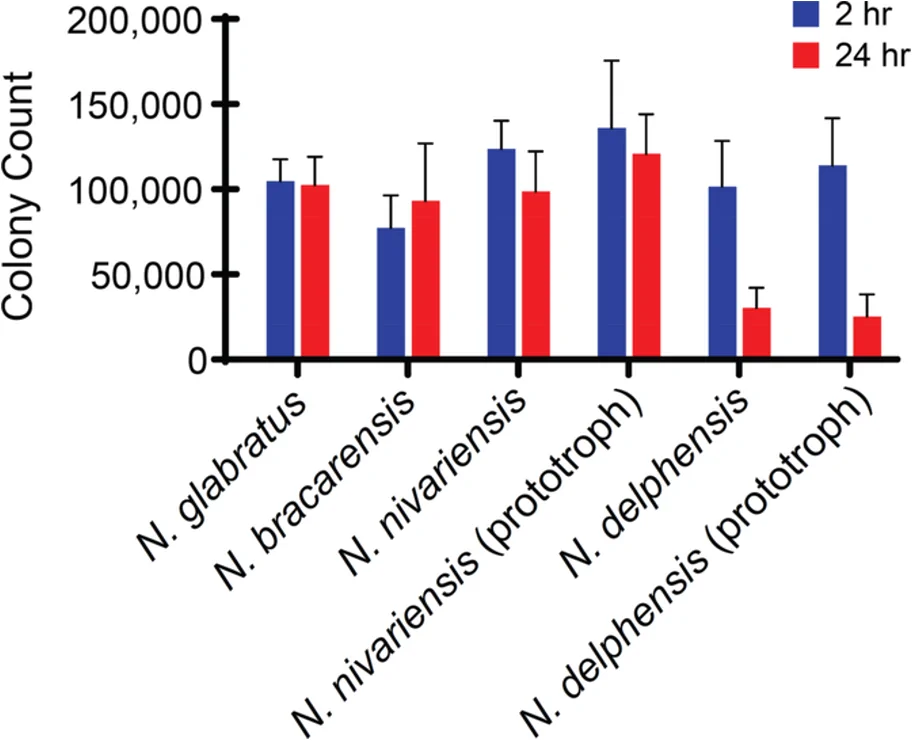
Ability of *Nakaseomyces* species to persist in a mouse macrophage survival model. Mouse macrophage cells were incubated with *Nakaseomyces* yeast cells, which are engulfed by the macrophages during phagocytosis. Macrophage cells are lysed and the number of yeast cells present at 2 h (representing the total number of engulfed yeast cells) and 24 h after phagocytosis (the number of yeast cells that survived) are quantified by plating on YEPD. The *his3ura3* auxotrophic strains were used for all species except *N. nivariensis* and *N. delphensis*, for which the prototrophic strain was also included. Bars represented the mean and standard deviation of three replicates.

Using a previously characterized persistence assay in mouse macrophages (Pezak et al. 2024), we determined the ability of each species to survive in macrophages as a proxy for pathogenicity (Figure 7) as all of the species except *N. delphensis* have been isolated from clinical samples. Examining persistence in mouse macrophage cells demonstrates that all of the species behave similar to *N. glabratus*, except *N. delphensis*. To confirm that *N. delphensis* was not being cleared as a result of the auxotrophies we introduced, we repeated the assay with the wild‐type *N. delphensis* and *N. nivariensis* and saw the same result, eliminating the likelihood that this clearance of fungal cells is because the cells are auxotrophic. It is unclear if that is a consequence of *N. delphensis* growing slower at 37°C relative to the other species (Figure 2B), but the data match clinical situations (Angoulvant et al. 2015; Legrand 2016; Zhou Li et al. 2021).

## 4 Discussion

Here, we have developed genetic tools to allow for the shuttling of genes between four *Nakaseomyces* species. The introduction of stable plasmids has allowed us to confirm that expression of *ScTHI5* is sufficient to confer thiamine prototrophy in the four species, and expression of *ScSNZ1* is sufficient to confer pyridoxine prototrophy, although *N. nivariensis* cells exhibit poor growth with plasmids containing these genes. Additionally, we confirm that *THI4*, which encodes one of the most highly expressed genes during thiamine starvation, is upregulated in each of the *Nakaseomyces* species. This indicates that even though they have lost the ability to make thiamine, these species still have the ability to sense thiamine concentrations and change gene expression.

Using CRISPR/Cas9, we have developed auxotrophic strains and determined the resolution of the those cuts to generate frameshift mutations in biosynthetic genes. This work builds on the previous work of transformation and CRISPR/Cas9 manipulations in this genus (Enkler et al. 2016; Zhou Li et al. 2021; Métivier et al. 2024). This allowed us to then introduce plasmids into these strains. Although it is not presented in this manuscript, we were able to delete *LEU2* in *N. nivariensis* with a nourseothricin resistance cassette, but only when we introduced 500 bp of homology on each side of the cassette. Furthermore, we were only able to identify one correct deletion in ~80 colonies. This is consistent with our observations that transformation efficiencies are lower in the non‐*glabratus* species and suggests that homologous recombination is not as efficient as in *N. glabratus*. However, other explanations are possible too. We believe that optimization of transformation and coupling repair templates with CRISPR/Cas9 targets will likely improve the efficiency of generating specifically tailored strains. The ability to introduce plasmids and maintain them allows for a lot of flexibility in comparative genomic analyses.

Our work indicates some interesting contrasts. We find that *Nakaseomyces ADH1* promoters express poorly in *S. cerevisiae*. It is possible that some of our results are a consequence of plasmid copy number maintenance. Figure 5A suggests that plasmid copy number is not a significant issue across the *Nakaseomyces* species for expression as native *ADH1* promoters express at roughly the same levels in each species on a plasmid. However, we have not quantified plasmid copy number, and it is possible that plasmids optimized for each species will generate different results.

Finally, generation of these genetic models allows us to ask questions about the *NgPMU2* and *NgPMU3* promoters, which are regulated by external phosphate and thiamine concentrations, respectively. These promoters are essentially non‐functional in *S. cerevisiae*, indicating an incompatibility between the transcription factors and the newly evolved promoters. However, examining *Nakaseomyces* species allows us to ask if these promoters will function in more closely related species. We show that both *de novo* promoters behave similarly in *N. glabratus* and *N. bracarensis*, suggesting both species have the *trans* and *cis* elements required for transcriptional upregulation in response to phosphate and thiamine starvation (Figure 6C). In contrast, *N. nivariensis* and *N. delphensis* only minimally upregulate *NgPMU2* and *NgPMU3* promoters, indicating that the *trans* machinery cannot recognize the *cis* elements in the promoters. These results do not indicate that the THI and PHO pathways are non‐functional in *N. nivariensis* and *N. delphensis*, just these new promoters are not able to be regulated in the two species. Here, we have presented a proof of concept, but a more detailed dissection of *trans* factors and *cis* elements can now be performed.

## 5 Conclusions

We have used CRISPR/Cas9 to generate more molecular genetic tools in four *Nakaseomyces* species. This work indicates that *N. glabratus* and *N. bracarensis* have many similarities in their transcriptional regulation, whereas *N. delphensis* and *N. nivariensis* appear to be more divergent. Generating these model systems provides more tools for researchers to manipulate four species that have different niches.

## Author Contributions

**Maria Abraham:** data collection, data analysis, original draft writing. **Christine L. Iosue:** conceptualization, data collection, data visualization, writing – editing. **Dennis D. Wykoff:** conceptualization, supervision, funding acquisition, writing – editing.

## Supporting information


**Figure S1:** Histograms of *ADH1*pr‐YFP expression in its native species.

**Table S1:** Strains used in this study.**Table S2:** Primers used in this study.**Table S3:** Efficiency of gRNAs in generating auxotrophy.

## Data Availability

Strains and plasmids are available upon request.
