# Supplementary material for: Genetic Tools in the Nakaseomyces clade for Evolutionary Comparisons of Signal Transduction Pathways
Source: Yeast. 2026 Jun 3;43(5):202–13. doi: 10.1002/yea.70029 (PMC13548925; doi:10.1002/yea.70029)
Supplement: Supplementary file 1 [file YEA-43-202-s002.pdf]

**Supplementary Table 1. Strains used in this study.**

| Strain | Genotype | Reference |
|---|---|---|
| | **Yeast strains** | |
| DC3 | *Saccharomyces cerevisiae* (K699 ade2-1 trp1-1 can 1-100 leu2-3,112 his3-11,15 ura3 GAL+) | Wykoff and O'Shea 2001 |
| DC338 | *Saccharomyces cerevisiae* SK1 (EY1081) | Gift of Angelika Amon strain 4095 |
| DG5 | *Nakaseomyces glabratus* his3- | Cormack and Falkow 1999 |
| DG176 | *Nakaseomyces glabratus* his3- ura3- | Kerwin and Wykoff 2012 |
| DY29 | *Nakaseomyces bracarensis* | Gift of Bin He |
| DY11 | *Nakaseomyces nivariensis* | Gift of Bin He |
| DY13 | *Nakaseomyces delphensis* | ATCC 24205 |
| DY23 | *Nakaseomyces bracarensis* ura3- | This study |
| DY24 | *Nakaseomyces nivariensis* ura3- | This study |
| DY25 | *Nakaseomyces delphensis* ura3- | This study |
| DY30 | *Nakaseomyces bracarensis* ura3- his3- | This study |
| DY31 | *Nakaseomyces nivariensis* ura3- his3- | This study |
| DY32 | *Nakaseomyces delphensis* ura3- his3- | This study |
| | **Plasmids** | |
| DB544 | pV1435: Solo vector pV1382 + sg*NgADE2* with *Ca* Cas9 | Addgene 111439 |
| DB576 | pV1435 with *Pac* I site upstream of *NgADE2* gRNA | This study |
| DB577 | DB576 + *NgADE2* gRNA, removing the PacI site | This study |
| DB592 | DB576 + *N. glabratus LEU2* gRNA (#2: primers O3766/O3767) | This study |
| DB617 | DB576 + *N. bracarensis HIS3* gRNA (#1: primers O3778/O3779) | This study |
| DB618 | DB576 + *N. nivariensis HIS3* gRNA (#2: primers O3823/O3824) | This study |
| DB619 | DB576 + *N. delphensis HIS3* gRNA (#1: primers O3826/3837) | This study |
| DB341 | *NgPGK1* pr-*ScTHI5* -pRS313 | This study |
| DB692 | *ScPGK1* pr-*ScSNZ1* -pRS313 | This study |
| DB549 | *ScSNO1* /*ScSNZ1* -pRS313 | This study |
| DB164 | *S. cerevisiae ADH1* pr-YFP-pRS313 | Iosue et al. 2016 |
| DB345 | *N. glabratus ADH1* pr-YFP-pRS313 | Iosue et al. 2016 |
| DB735 | *N. bracarensis ADH1* pr-YFP-pRS313 | This study |
| DB726 | *N. nivariensis ADH1* pr-YFP-pRS313 | This study |
| DB727 | *N. delphensis ADH1* pr-YFP-pRS313 | This study |
| DB266 | *S. cerevisiae THI4* pr-YFP-pRS313 | Iosue et al. 2020 |
| DB267 | *N. glabratus THI4* pr-YFP-pRS313 | Iosue et al. 2020 |
| DB730 | *N. bracarensis THI4* pr-YFP-pRS313 | This study |
| DB728 | *N. nivariensis* THI4pr-YFP-pRS313 | This study |
| DB729 | *N. delphensis THI4* pr-YFP-pRS313 | This study |
| DB412 | *N. glabratus PMU2* pr-YFP-pRS313 | Nahas et al. 2018 |
| DB347 | *N. glabratus PMU3* pr-YFP-pRS313 | Nahas et al. 2018 |

**Supplementary Table 2. Primers used in this study.**

| Purpose | Primer number | Primer sequence |
|---|---|---|
| Engineer *Pac* I site upstream of gRNA in pV1435 (plasmid to be named DB576) | O3620 | CAGTGAAAGtTAAtTaATCGgttgggccgtatgattgtcg |
| | O3621 | ggcccaacCGATtAaTTAaCTTTCACTGCGGAGAAGTTTC |
| check for *NgADE2* gRNA | O3540 | GGTCAAACGCTGTAGAAGTG |
| | O3541 | GGCAAACCCGCGGTGGCGGC |
| DB576 + *NgADE2* gRNA, removing the *Pac* I site | O3628 | TCGAAACTTCTCCGCAGTGAAAGATAAATGATCGgttgggccgtatgattgtcgGTTTTAGAGCTAGAAATAGCAAGTT |
| | O3629 | AACTTGCTATTTCTAGCTCTAAAACcgacaatcatacggcccaacCGATCATTTATCTTTCACTGCGGAGAAGTTTCGA |
| DB576 + *NgLEU2* gRNA #1 | O3763 | TCGAAACTTCTCCGCAGTGAAAGATAAATGATCGgttgttagagaactagtgggGTTTTAGAGCTAGAAATAGCAAGTT |
| | O3764 | AACTTGCTATTTCTAGCTCTAAAACcccactagttctctaacaacCGATCATTTATCTTTCACTGCGGAGAAGTTTCGA |
| check for *NgLEU2* gRNA #1 (with O3730) | O3765 | gttgttagagaactagtggg |
| **DB576 + *NgLEU2* gRNA #2 (DB592 in Supp. Table 1: used in this study)*** | **O3766** | **TCGAAACTTCTCCGCAGTGAAAGATAAATGATCGggcgctgtcagaccagaacaGTTTTAGAGCTAGAAATAGCAAGTT** |
| | **O3767** | **AACTTGCTATTTCTAGCTCTAAAACtgttctggtctgacagcgccCGATCATTTATCTTTCACTGCGGAGAAGTTTCGA** |
| check for *NgLEU2* gRNA #2 (with O3730) | O3768 | ggcgctgtcagaccagaaca |
| check for new gRNA - pair with specific gRNA primer | O3730 | CCGTTCATCTCTTCACCAGG |
| PCR and sequence *NgLEU2* gRNA locus to confirm frameshift mutation | O3785 | TGATGCCGTGCTTCTGGGTG |
| | O3786 | TTACATGGTCTTAGATTAGC |
| **DB576 + *N. bracarensis HIS3* gRNA #1 (DB617 in Supp. Table 1: used in this study)*** | **O3778** | **TCGAAACTTCTCCGCAGTGAAAGATAAATGATCGgaagcactccgggtggtcgcGTTTTAGAGCTAGAAATAGCAAGTT** |
| | **O3779** | **AACTTGCTATTTCTAGCTCTAAAACgcgaccacccggagtgcttcCGATCATTTATCTTTCACTGCGGAGAAGTTTCGA** |
| check for *N. bracarensis HIS3* gRNA #1 (with O3730) | O3780 | gaagcactccgggtggtcgc |
| DB576 + *N. bracarensis HIS3* gRNA #2 | O3781 | TCGAAACTTCTCCGCAGTGAAAGATAAATGATCGcgacgaccaccacacggtcgGTTTTAGAGCTAGAAATAGCAAGTT |
| | O3782 | AACTTGCTATTTCTAGCTCTAAAACcgaccgtgtggtggtcgtcgCGATCATTTATCTTTCACTGCGGAGAAGTTTCGA |
| check for *N. bracarensis HIS3* gRNA #2 (with O3730) | O3783 | cgacgaccaccacacggtcg |
| PCR and sequence *NbHIS3* gRNA locus to confirm frameshift mutation | O2782 | ATGCACCGCAGTTGTATG |
| | O2783 | CTATGCTAGCACACCCTTGG |
| DB576 + *N. nivariensis HIS3* gRNA #1 | O3820 | TCGAAACTTCTCCGCAGTGAAAGATAAATGATCGcggtggtgtagtgaccttacGTTTTAGAGCTAGAAATAGCAAGTT |
| | O3821 | AACTTGCTATTTCTAGCTCTAAAACgtaaggtcactacaccaccgCGATCATTTATCTTTCACTGCGGAGAAGTTTCGA |
| check for *N. nivariensis HIS3* gRNA #1 (with O3730) | O3822 | cggtggtgtagtgaccttac |
| **DB576 + *N. nivariensis HIS3* gRNA #2 (DB618 in Supp. Table 1: used in this study)*** | **O3823** | **TCGAAACTTCTCCGCAGTGAAAGATAAATGATCGaaggagtatcctgccagttcGTTTTAGAGCTAGAAATAGCAAGTT** |
| | **O3824** | **AACTTGCTATTTCTAGCTCTAAAACgaactggcaggatactccttCGATCATTTATCTTTCACTGCGGAGAAGTTTCGA** |
| check for *N. nivariensis HIS3* gRNA #2 (with O3730) | O3825 | aaggagtatcctgccagttc |
| PCR *NnHIS3* gRNA locus to confirm frameshift mutation | O4138 | ATGTCTGAGCATACTGCTTTTATC |
| | O4139 | CAGACAATCAACATGCATTG |
| Sequence *NnHIS3* gRNA locus to confirm frameshift mutation | O4140 | AGCCCCAACTCCACTACGGC |
| **DB576 + *N. delphensis HIS3* gRNA #1 (DB619 in Supp. Table 1: used in this study)*** | **O3826** | **TCGAAACTTCTCCGCAGTGAAAGATAAATGATCGcaggtggctctgtcacttgaGTTTTAGAGCTAGAAATAGCAAGTT** |
| | **O3827** | **AACTTGCTATTTCTAGCTCTAAAACtcaagtgacagagccacctgCGATCATTTATCTTTCACTGCGGAGAAGTTTCGA** |
| check for *N. delphensis HIS3* gRNA #1 (with O3730) | O3828 | caggtggctctgtcacttga |
| DB576 + *N. delphensis HIS3* gRNA #2 | O3829 | TCGAAACTTCTCCGCAGTGAAAGATAAATGATCGcagagtactccttgcctaacGTTTTAGAGCTAGAAATAGCAAGTT |
| | O3830 | AACTTGCTATTTCTAGCTCTAAAACgttaggcaaggagtactctgCGATCATTTATCTTTCACTGCGGAGAAGTTTCGA |
| check for *N. delphensis HIS3* gRNA #2 (with O3730) | O3831 | cagagtactccttgcctaac |
| PCR *NdHIS3* gRNA locus to confirm frameshift mutation | O4135 | ATGGACGAACACTTTGCTTTTAT |
| | O4136 | ACGTTTCAGTCCCAGCTCCACC |
| Sequence *NdHIS3* gRNA locus to confirm frameshift mutation | O4137 | CTCTCGACAGCGCCTCGTCC |
| *PCR NATMX6* to delete *N. nivariensis LEU2* | O4009 | ATGACTACCAAAAATATCGTcggatccccgggttaattaa |
| | O4010 | CAGCTTTTGCAACTGCATCAgaattcgagctcgtttaaac |
| 500 bp homology upstream of *NnLEU2* (for deletion of *NnLEU2*) | O4007 | tgtgttatcattcactaacc |
| | O4008 | tccgACGATATTTTTGGTAGTCAT |
| 500 bp homology downstream of *NnLEU2* (for deletion of *NnLEU2*) | O4011 | TGATGCAGTTGCAAAAGCTG |
| | O4012 | ttgcgcatttattcaagaag |
| check for *Nnleu2 Δ NATMX6* | O4013 | gtttttattaacttccatttttacc |
| | O780 | TTAATTAACCCGGGGATCCG |
| *NgPGK1* promoter | O1691 | gtgatcttgtctgtagacatTATCGAATAGATGTATGTATG |
| | O1665 | gagctccaccgcggtggcggccgctctagaactaTCTGGTTCCCGCTATAAGATG |
| *ScTHI5* ORF | O1692 | CATACATACATCTATTCGATAatgtctacagacaagatcac |
| | O1693 | gtatcgataagcttgatatcgaattcctgcagcccgggTTGAGCATTAAATTAACACC |
| check primers for *ScTHI5* | O1043 | ATTGGAAGAGTACTTGGCCA |
| | O1044 | TGAAAGTACCTTCCTGTCTG |
| *ScPGK1* promoter | O3858 | aattggagctccaccgcggtggcggccgctctagaactagTCTTGTTTTGCAAGTACCAC |
| | O3859 | ttaaagtcttctccagtcatTGTTTTATATTTGTTGTAAAAAG |
| *ScSNZ1* ORF | O3860 | CTTTTTACAACAAATATAAAACAatgactggagaagactttaa |
| | O3861 | acggtatcgataagcttgatatcgaattcctgcagcccggGTAAATAATATCCAGCATAC |
| check primers for *ScPGK1* promoter | O3862 | tgaattgtaatacgactcac |
| | O3863 | CTCACTCTTTCCTTACTCAC |
| check primers for *ScSNZ1* | O3604 | GGAGATAAAGGCGTGCCAAC |
| | O3605 | GTAGCCTCTACTACTGCAGTGG |
| *ScSNO1* /*ScSNZ1* promoter and ORF | O3388 | ggagctccaccgcggtggcggccgctctagaactagtTTTGAACCATATCAAGCTAC |
| | O3389 | cggtatcgataagcttgatatcgaattcctgcagcccgggTCTAGTAAATAATATCCAGC |
| check primers for *ScSNO1* /*ScSNZ1* | T7 | TAATACGACTCACTATAGGG |
| | O3513 | GATACAAGATTTCATGATTGG |

| Purpose | Primer number | Primer sequence |
|---|---|---|
| *ScADH1* promoter | O808 | ggagctccaccgcggtggcggccgctctagaactagtGGTTGCTACCAGTATAAATAG |
| | O809 | cagtgaataattcttcacctttagacat**GGATCC**TGTATATGAGATAGTTGATTG |
| *N. glabratus ADH1* promoter | O1526 | ggtggcggccgctctagaactagtggatccCGGTCCTAGTGTTCATACTG |
| | O1527 | **accagtgaataattcttcacctttagacat**tgtttatgtgttttttgcag |
| *N. bracarensis ADH1* promoter | O4335 | GCTCCACCGCGGTGGCGGCCGCTCTAGAACTAGTGGATCC**GACGACAATCAGGGGTTGTG** |
| | O4336 | TTGGGACAACACCAGTGAATAATTCTTCACCTTTAGAcatTTTTTGTTGTTTTTTGTTGT |
| check primers for *NbADH1* pr-YFP | O4337 | CCAGAACCAGAACCATAACC |
| | O245 | GGGACAACACCAGTGAATAATTCTTCACC |
| *N. nivariensis ADH1* promoter | O4020 | GCTCCACCGCGGTGGCGGCCGCTCTAGAACTAGTGGATCCggaacggttgctttctcgag |
| | O4021 | TTGGGACAACACCAGTGAATAATTCTTCACCTTTAGAcattattgatatgtatttatttt |
| check primers for *NnADH1* pr-YFP (with O245) | O4022a | tactgcaatgagcaaggtgg |
| *N. delphensis ADH1* promoter | O4023 | GCTCCACCGCGGTGGCGGCCGCTCTAGAACTAGTGGATCCaaaggttactctccgcgtag |
| | O4024 | TTGGGACAACACCAGTGAATAATTCTTCACCTTTAGAcattgttttattgtttttgtatt |
| check primer for *NdADH1* pr-YFP (with O245) | O4025a | gtagaccaaacttgctctac |
| *N. bracarensis THI4* promoter | O3296 | caccgcggtggcggccgctctagaactagtggatcc**agtatatttcaagttctccg** |
| | O3297 | caccaccagtgaataattcttcacctttagacat**ATTTTTTTATTTTTATTTTTACC** |
| check primer for *NbTHI4* pr-YFP (with O245) | O3298 | atatcaaatagaggatagtc |
| *N. nivariensis THI4* promoter | O4026 | GCTCCACCGCGGTGGCGGCCGCTCTAGAACTAGTGGATCCagaaaaaaataaattcgtgc |
| | O4027 | TTGGGACAACACCAGTGAATAATTCTTCACCTTTAGAcattataatatattttatttttt |
| check primer for *NnTHI4* pr-YFP (with O245) | O4028a | atactcaactttgaatgtgc |
| *N. delphensis THI4* promoter | O4029 | GCTCCACCGCGGTGGCGGCCGCTCTAGAACTAGTGGATCCaagtgaaaatagccgccgag |
| | O4030 | TTGGGACAACACCAGTGAATAATTCTTCACCTTTAGAcatttttttttcaggctgttatta |
| check primer for *NdTHI4* pr-YFP (with O245) | O4031a | tgcatgagcaagaaagacag |

*Primers in bold were used to generate strains in this study.

## Supplementary Table 3. Efficiency of gRNAs in generating auxotrophy

| | | Auxotrophic colonies | Total colonies | % Auxotrophic |
|---|---|---|---|---|
| ***N. glabratus leu2-*** | gRNA #1 | 4 | 4 | 100 |
| | gRNA #2* | 6 | 17 | 35 |
| ***N. bracarensis his3-*** | gRNA #1* | 2 | 2 | 100 |
| | gRNA #2 | 0 | 10 | 0 |
| ***N. nivariensis his3-*** | gRNA #1 | 0 | 37 | 0 |
| | gRNA #2* | 2 | 10 | 20 |
| ***N. delphensis his3-*** | gRNA #1* | 1 | 8 | 13 |
| | gRNA #2 | 0 | 3 | 0 |

*gRNA used to generate strains used in this study.
