# Supplementary material for: Genetic Tools in the Nakaseomyces clade for Evolutionary Comparisons of Signal Transduction Pathways
Source: Yeast. 2026 Jun 3;43(5):202–13. doi: 10.1002/yea.70029 (PMC13548925; doi:10.1002/yea.70029)
Supplement: Supplementary file 2 [file YEA-43-202-s001.docx]

Supplemental Figure S1

S. cerevisiae | *N. glabratus* | *N. bracarensis* | *N. nivariensis* | *N. delphensis*

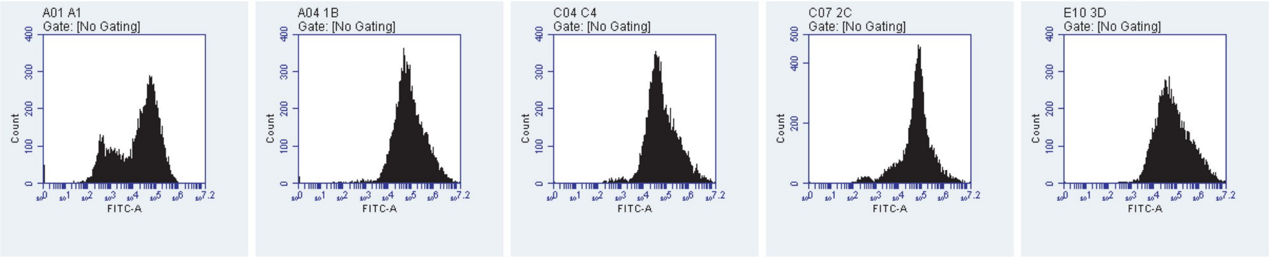

*ScADH1*pr-YFP | *NgADH1*pr-YFP | *NbADH1*pr-YFP | *NnADH1*pr-YFP | *NdADH1*pr-YFP
